# Anti-mucin 1 chimeric antigen receptor T cells for adoptive T cell therapy of cholangiocarcinoma

**DOI:** 10.1038/s41598-021-85747-9

**Published:** 2021-03-18

**Authors:** Kamonlapat Supimon, Thanich Sangsuwannukul, Jatuporn Sujjitjoon, Nattaporn Phanthaphol, Thaweesak Chieochansin, Naravat Poungvarin, Sopit Wongkham, Mutita Junking, Pa-thai Yenchitsomanus

**Affiliations:** 1 Siriraj Center of Research Excellence for Cancer Immunotherapy (SiCORE-CIT), Research Department, Faculty of Medicine Siriraj Hospital, Mahidol University, Bangkok, Thailand; 2 Graduate Program in Immunology, Department of Immunology, Faculty of Medicine Siriraj Hospital, Mahidol University, Bangkok, Thailand; 3 Division of Molecular Medicine, Research Department, Faculty of Medicine Siriraj Hospital, Mahidol University, 2 Wanglang Road, Bangkoknoi, Bangkok, Thailand; 4 Department of Clinical Pathology, Faculty of Medicine Siriraj Hospital, Mahidol University, Bangkok, Thailand; 5 Department of Biochemistry, and Center for Translational Medicine, Faculty of Medicine, Khon Kaen University, Khon Kaen, Thailand; 6 Cholangiocarcinoma Research Institute, Khon Kaen University, Khon Kaen, Thailand

**Keywords:** Cancer therapy, Cancer, Immunology, Immunotherapy

## Abstract

Current treatments for cholangiocarcinoma (CCA) are largely unsuccessful due to late diagnosis at advanced stage, leading to high mortality rate. Consequently, improved therapeutic approaches are urgently needed. Chimeric antigen receptor (CAR) T cell therapy is a newly potential therapy that can recognize specific surface antigen without major histocompatibility complex (MHC) restriction. Mucin 1 (MUC1) is an attractive candidate antigen as it is highly expressed and associated with poor prognosis and survival in CCA. We, therefore, set forth to create the fourth-generation CAR (CAR4) construct containing anti-MUC1-single-chain variable fragment (scFv) and three co-stimulatory domains (CD28, CD137, and CD27) linked to CD3ζ and evaluate anti-MUC1-CAR4 T cells in CCA models. Compared to untransduced T cells, anti-MUC1-CAR4 T cells produced increased levels of TNF-α, IFN-γ and granzyme B when exposed to MUC1-expressing KKU-100 and KKU-213A CCA cells (all *p* < 0.05). Anti-MUC1-CAR4 T cells demonstrated specific killing activity against KKU-100 (45.88 ± 7.45%, *p* < 0.05) and KKU-213A cells (66.03 ± 3.14%, *p* < 0.001) at an effector to target ratio of 5:1, but demonstrated negligible cytolytic activity against immortal cholangiocytes. Furthermore, the anti-MUC1-CAR4 T cells could effectively disrupt KKU-213A spheroids. These activities of anti-MUC1-CAR4 T cells supports the development of this approach as an adoptive T cell therapeutic strategy for CCA.

## Introduction

Cholangiocarcinoma (CCA) is cancer of the biliary tree—extremely challenging to treat. Standard treatments are generally unsuccessful in advanced-stage^1^. The 5-year survival rate after surgical resection is low (23–63%), with high recurrence rate^2^. For unresectable patients, chemotherapy is a palliative treatment modality, with poor response rate and side effects^3^. Targeted therapy drug, such as infigratinib, is restricted to a subgroup of patients with advanced/metastatic CCA with *FGFR2* translocations^4^. Therefore, new therapeutic approaches are urgently required.

Immunotherapies have been reported for CCA treatment^1^. Treatments of monoclonal antibodies^5^, lysate-pulsed dendritic cells (DCs) combined with ex vivo activated T cells^6^ and CD4^+^ tumor-infiltrating lymphocytes (TILs)^7^, were used for CCA patient. Although, these approaches are susceptible to MHC downregulation and therapeutic failure^8^. Adoptive T cell therapy using chimeric antigen receptor (CAR) T cells is an alternative strategy for cancer therapy with high efficacy in hematological malignancies. CARs are synthetic fusions that recognize surface cancer antigens and enable engineered T cells to induce cancer cell apoptosis without MHC recognition^9^.

CAR T cells showed impressive efficacy against relapsed or refractory B-cell acute lymphoblastic leukemia (ALL), and CD19-CAR T cells have now been approved^10^. However, the anti-tumor effect of CAR T cells is less evident in solid tumors^9^. Generally, three generations of CAR T cells against cancers have been produced. Among these, the common structure of CAR included antigen recognition, spacer, transmembrane, and intracellular domains. The intracellular domain of each generation of CAR contained different numbers of co-stimulatory molecules linked to CD3ζ. The second- and third-generations of CAR T cells demonstrated higher anti-tumor effects compared to the first-generation^11,12^. The fourth-generation CAR (CAR4) T cells in the previous studies, which refers to as ‘T cells redirected for antigen‐unrestricted cytokine‐initiated killing (TRUCKS)’ or ‘Armored CAR T cells’, are modified T cells to co-express the CAR molecule and ligands or cytokines to enhance T cell function^13^. Here, we proposed an alternative version of fourth-generation CAR T cells consisting of three costimulatory molecules (CD28, CD137, and CD27) linked to CD3ζ. Notably, our group has recently reported the fourth-generation CAR T cells to target CD133-expressing and folate receptor alpha-expressing cancer models^14,15^.

Selection of suitable antigens on cancer cells is essential for designing an effective CAR T cell approach and avoidance of side effects. Among several cancer antigens, mucin 1 (MUC1)—a type I transmembrane protein that plays role in mucous membrane protection, signal transduction, and modulation of immune system^16^, is one of the best potential target antigens for CAR T cell therapy because it is overexpressed in several cancers and its expression is related to cancer progression^17^. This potential target antigen was also found to be expressed in 50–86.5% of CCA tissues among patients^18–20^. Interestingly, the cancer-associated MUC1 is hypoglycosylated, compared to the heavily glycosylated form found in normal cells, so it can be specifically targeted by CAR T cells without on-target off-tumor effect^21^.

Several studies have generated and modified CAR T cells targeting MUC1, and have investigated their functional efficacies in several cancer models^11,12,22–24^. The use of these CAR T cells was also reported from some clinical trials^25^. To date, none of these CAR T cells has been approved for clinical use. The anti-tumor efficacy of MUC1-targeting CAR T cells was studied against breast cancer by comparing the single chain variable fragment (scFv), spacer length, and generations of CAR^11^. High efficacy of hypoglycosylated MUC1-targeting CAR T cells was demonstrated in triple negative breast cancer model (TNBC)^23^. Also, in hematological and pancreatic cancers, CAR T cells targeting hypoglycosylated MUC1 showed cancer regression^22^. Other developments include the description of MUC1-CAR T cells with IL-22 secretion in head and neck squamous cell carcinoma (HNSCC)^24^ or an inverted cytokine receptor (IL-4/IL-7) in breast cancer model^12^. These studies illustrated impressive anti-tumor functions of anti-MUC1-CAR T cells against several cancers. However, activity of anti-MUC1-CAR T cells in models of CCA has never been reported. This study set out to engineer anti-MUC1-CAR T cells and examine their anti-tumor effects against MUC1-expressing CCA cells. Our results provide encouragement for the application of anti-MUC1-CAR T cells in the immunotherapy of CCA.

## Results

### MUC1 was expressed in CCA tissues

**Figure 1:**
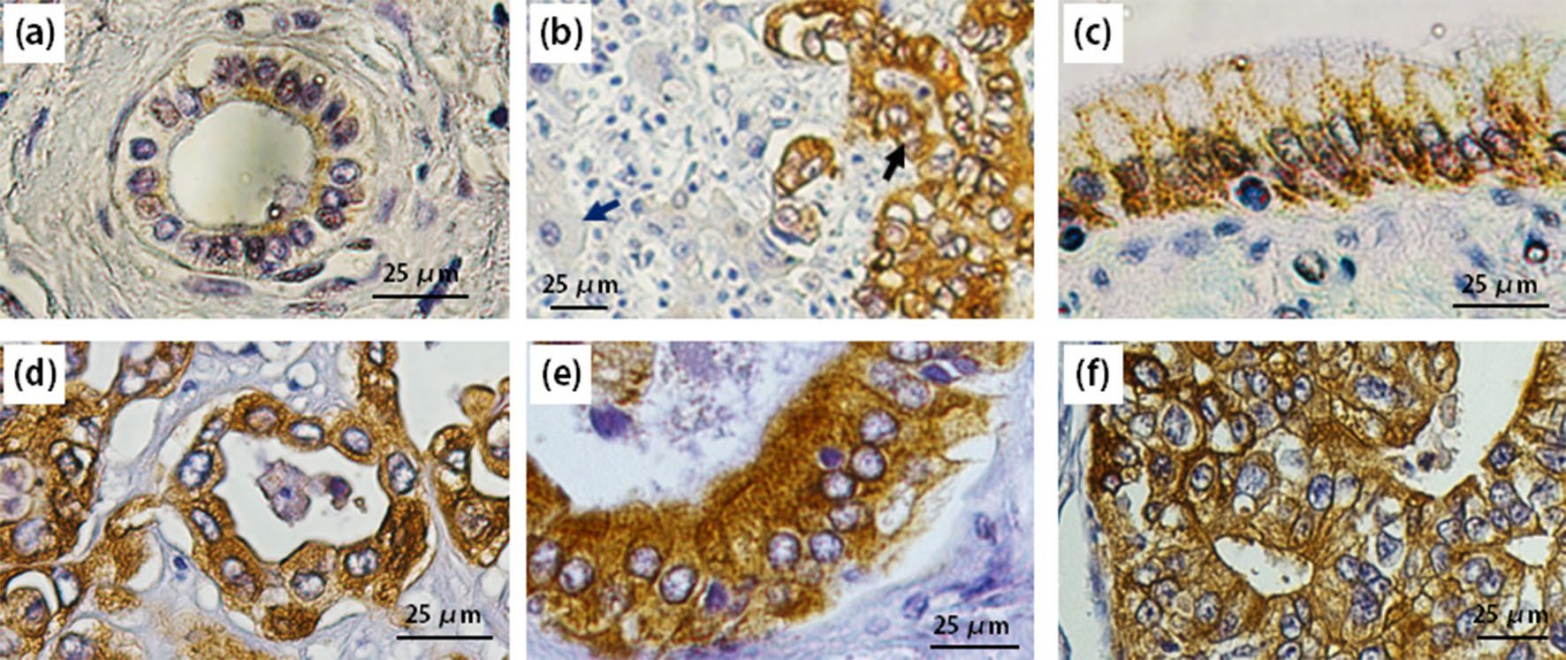
Expression of MUC1 protein in cholangiocarcinoma tissue samples. MUC1 protein in CCA was detected by immunohistochemistry (IHC). (**a**) Normal bile duct tissue. (**b**) The adjacent area of normal (liver) and cancerous (CCA) tissues (indicated by blue and black arrow, respectively). (**c**–**f**) Four tissue samples from four individual patients with CCA. In the positive areas, MUC1 were stained in both cytoplasm and on cell surface.

To examine MUC1 protein expression, four CCA tissue samples from Thai patients with CCA were analyzed by IHC. MUC1 was weakly expressed in adjacent normal bile duct cells (Fig. 1a), but was highly expressed in all four CCA tissues (Fig. 1c–f). MUC1 was found to localize in both the cytoplasm and membrane of the cancerous cells. In the tissue sample where the normal and cancerous tissues were adjacent, MUC1 was not detected in the nearby hepatocytes, but it was detected in the invading cancerous CCA cells (Fig. 1b).

### MUC1 was expressed in CCA cell lines

**Figure 2:**
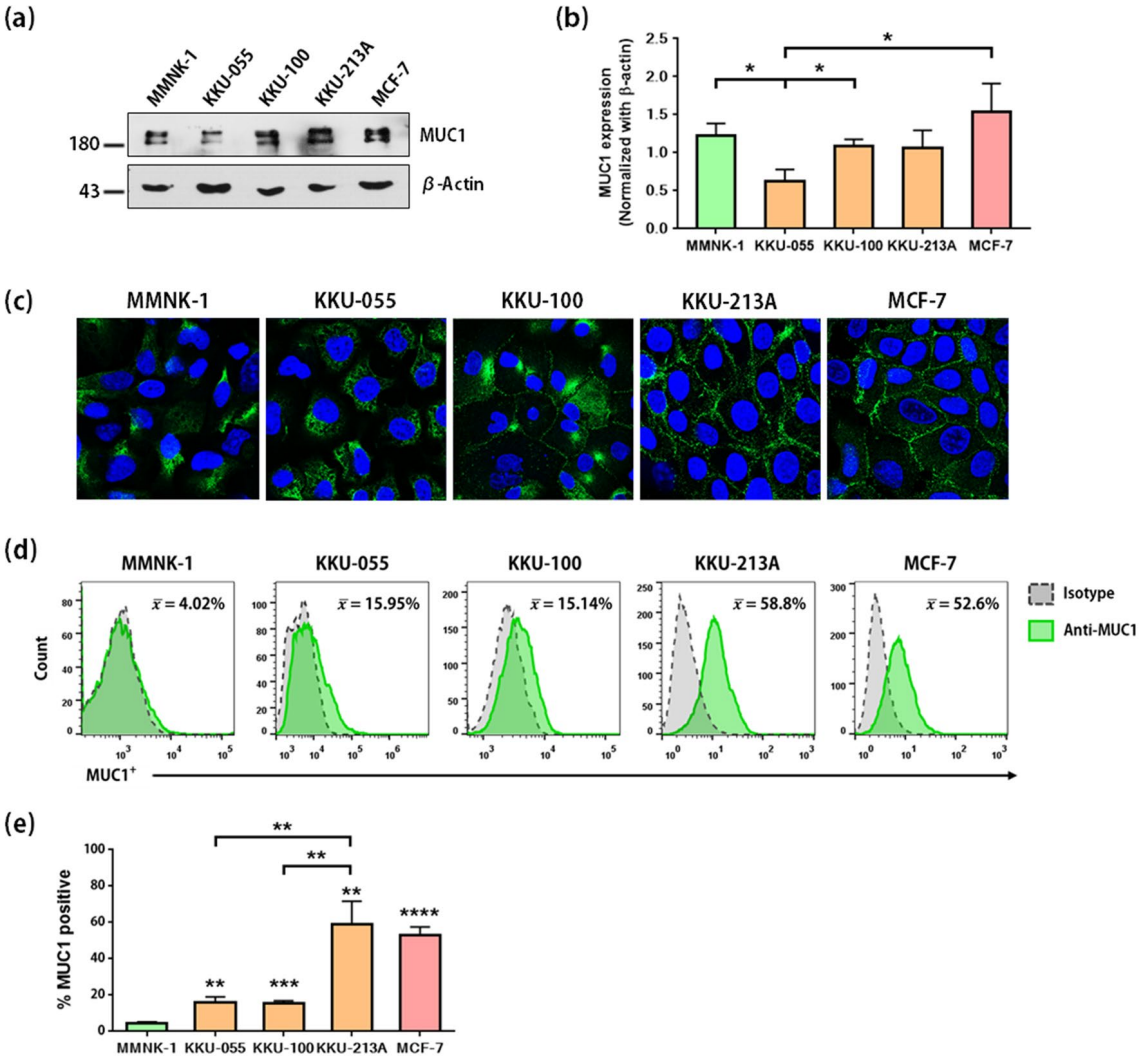
Expression of MUC1 protein in cholangiocarcinoma cell lines. (**a**) Representative immunoblot analysis showing total protein expression of MUC1 in cholangiocytes (MMNK-1), CCA cell lines (KKU-055, KKU-100, and KKU-213A), and a breast cancer cell line (MCF-7). The blots of cell lysate samples were cropped from different parts of the same gel. Full-length blots were presented in Supplementary Fig. 1. The MUC1 protein expression levels were normalized with β-actin. The data obtained from three independent experiments were plotted as bar graphs (**b**). Expression of MUC1 in cells was detected by immunofluorescence assay (IFA) (**c**), and cell surface expression of MUC1 was stained without permeabilization and examined by flow cytometry (**d**), which was plotted as bar graphs (**e**). All data with error bars (in **b** and **e**) represents the mean ± SD of three independent experiments. Staining with isotype control antibody was used as a negative control for IFA (data not shown) and flow cytometry. All data was analyzed by Student *t*-test (asterisks indicate statistically significant differences: *p < 0.05, **p < 0.01, ***p < 0.001 and ****p < 0.0001).

MUC1 expression in MMNK-1, KKU-055, KKU-100, KKU-213A, and MCF-7 cell lines was investigated. The result showed that MUC1 was expressed in all cell lines (Fig. 2a,b and Supplementary Fig. 1). MUC1 was previously reported to be highly expressed in MCF-7 cells^26^, which were used as positive control cells (Fig. 2a,b).

By IFA, cell surface expression of MUC1 was observed in all cancerous cell lines. In contrast, MUC1 expression was mostly located in the cytoplasm of MMNK-1 immortalized cholangiocytes, with low cell surface expression (Fig. 2c). In addition, MUC1 on the cells were stained without permeabilization. The results showed that MUC1 was strongly expressed on the surface of KKU-213A (58.81 ± 12.59%) and MCF-7 (52.58 ± 4.673%), intermediately expressed in KKU-055 (15.95 ± 2.843%) and KKU-100 (15.14 ± 1.526%), and weakly expressed in MMNK-1 cells (4.027 ± 0.994%) (Fig. 2d,e). The MUC1 expression on the membrane of KKU-055, KKU-100, KKU-213A, and MCF-7 was significantly higher than that of MMNK-1 (*p*-values 0.0024, 0.0005, 0.0017, and < 0.0001, respectively). Compared to KKU-055 and KKU-100, MUC1 expression on the surface of KKU-213A cells was also significantly higher (*p*-values 0.0045 and 0.0040, respectively). The cell surface expression of MUC1 on CCA cells indicates that it is a potential target for CAR T cell immunotherapy.

### Higher cytolytic function of the fourth-generation anti-MUC1-CAR T cells than that of the second-generation anti-MUC1-CAR T cells

**Figure 3:**
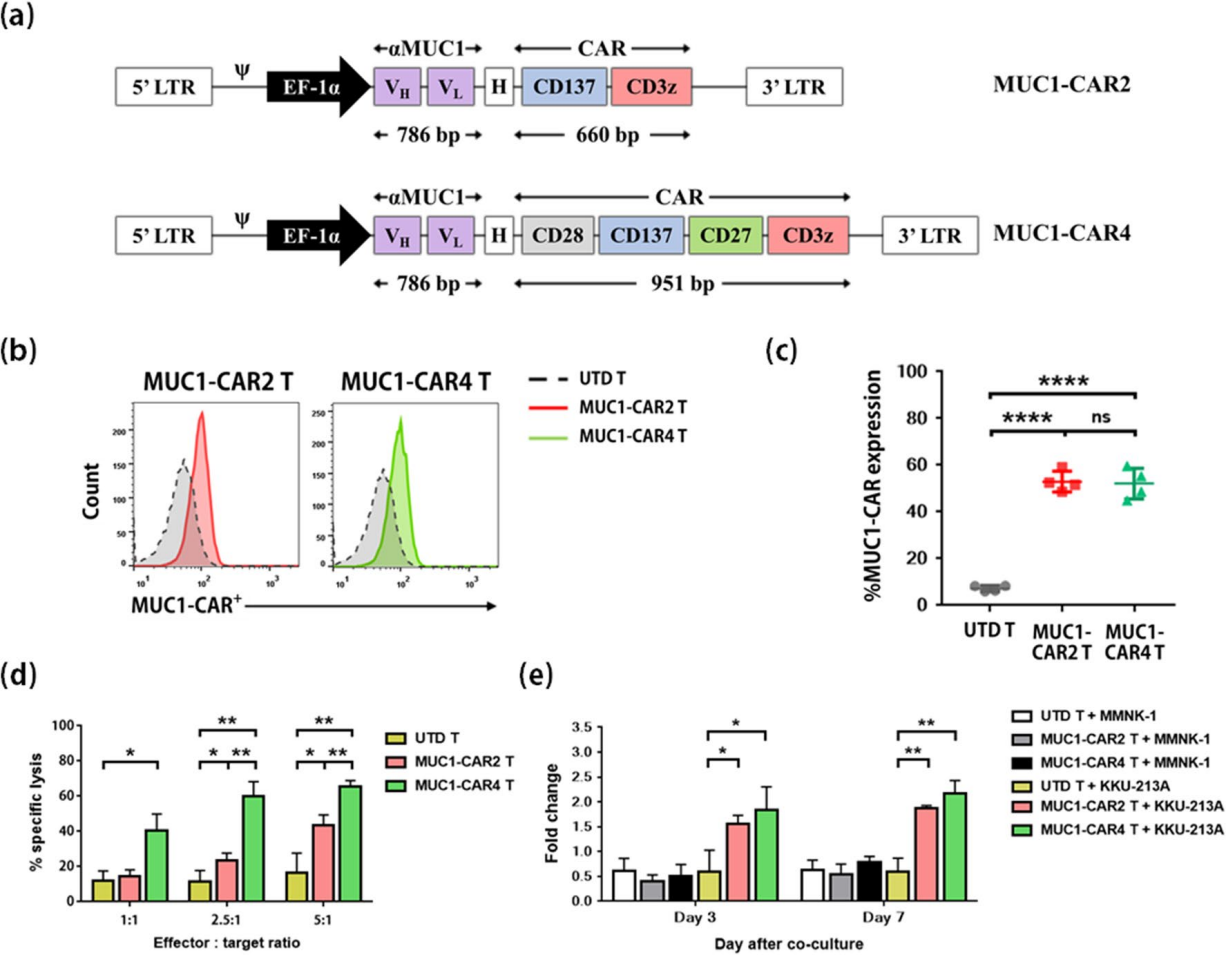
Anti-MUC1-CAR constructs, anti-MUC1-CAR T cell generation and their anti-tumor responses. (**a**) A schematic representation of the anti-MUC1-CAR2 (top) and anti-MUC1-CAR4 (bottom) lentiviral constructs. H represents hinge (spacer) domain. (**b**) Representative transduction efficiency of the untransduced T cells (UTD, gray), anti-MUC1-CAR2 T cells (red), and anti-MUC1-CAR4 T cells (green) detected by flow cytometry. (**c**) The transduction efficiency summarized from 4 independent experiments. (**d**) The bar graph represented percentages of KKU-213A cell lysis from 3 independent experiments. (**e**) T cell expansion after exposure to either MMNK-1 or KKU-213A cells. All data with error bars represents mean ± SD. Statistical differences were analyzed by Student *t*-test (asterisks indicate *p*-values: *p < 0.05, **p < 0.01, ***p < 0.001 and ****p < 0.0001).

A schematic drawing of the second-generation anti-MUC1-CAR (anti-MUC1-CAR2) and the fourth-generation anti-MUC1-CAR (anti-MUC1-CAR4) constructs were shown in Fig. 3a. The CAR protein expression was evaluated in HEK293T cells transfected with anti-MUC1-CAR plasmids. The results showed that the anti-MUC1-CAR2 and anti-MUC1-CAR4 proteins could be expressed from the anti-MUC1-CAR plasmids and presented on HEK293T cell surface, compared to untransfected cells (Supplementary Fig. 3a). The full translation of both CAR proteins was demonstrated by CD3ζ detection (Supplementary Fig. 3b–d).

To generate anti-MUC1-CAR T cells, PHA-activated lymphocytes were transduced with lentivirus-containing supernatant. Cell expansion after three days of PHA-activation is shown in Supplementary Fig. 4a. The transduction efficiency of anti-MUC1-CAR2 was 52.98 ± 4.393% and anti-MUC1-CAR4 expression was 52.13 ± 6.58%, compared to untransduced (UTD) T cells (both *p* < 0.0001) as detected by flow cytometry. The transduction efficiencies between anti-MUC1-CAR2 and anti-MUC1-CAR4 T cells were not significantly different (Fig. 3b,c).

To compare cytolytic functions of anti-MUC1-CAR2 T and anti-MUC1-CAR4 T cells, each generation of CAR T cells was co-cultured with the high MUC1-expressing CCA cells, KKU-213A. Both generations of CAR T cells demonstrated anti-tumor effects compared to UTD T cells. Nevertheless, the anti-MUC1-CAR4 T cells showed the highest killing activity (60.5 ± 8.22%), followed by the anti-MUC1-CAR2 T cells (23.71 ± 3.89%) and UTD T cells (11.69 ± 6.12%) at an effector to target (E:T) ratio of 2.5:1. Similarly, the percentages of specific lysis at an E:T ratio of 5:1 were 65.43 ± 3.56% for the anti-MUC1-CAR4 T, 43.66 ± 5.76% for anti-MUC1-CAR2 T, and 16.67 ± 11.07% for UTD T cells. The cytolytic activity of the anti-MUC1-CAR4 T cells was significantly higher than that of the anti-MUC1-CAR2 T cells at E:T ratios of 2.5:1 (*p*-value 0.0023) and 5:1 (*p*-value 0.0051) (Fig. 3d).

To examine the numbers of cell expansion, the anti-MUC1-CAR2 T and anti-MUC1-CAR4 T cells were co-cultured with MMNK-1 and KKU-213A cells. While there was no cellular expansion when the CAR T cells were co-cultured with MMNK-1 cells, significant MUC1-specific cellular expansions of anti-MUC1-CAR4 T cells and anti-MUC1-CAR2 T cells were observed, compared to UTD T cells (*p* < 0.05 at day 3 and *p* < 0.01 at day 7). The cellular expansion of the anti-MUC1-CAR4 T cells tended to be higher than that of the anti-MUC1-CAR2 T cells (Fig. 3e). Overall data suggested that the anti-MUC1-CAR4 T cells showed higher killing activity and cellular expansion; thus, the anti-MUC1-CAR4 T cells were further studied for anti-tumor activity against CCA cells.

### Phenotypes, activation and exhaustion markers, and memory subsets of anti-MUC1-CAR4 T cells

**Figure 4:**
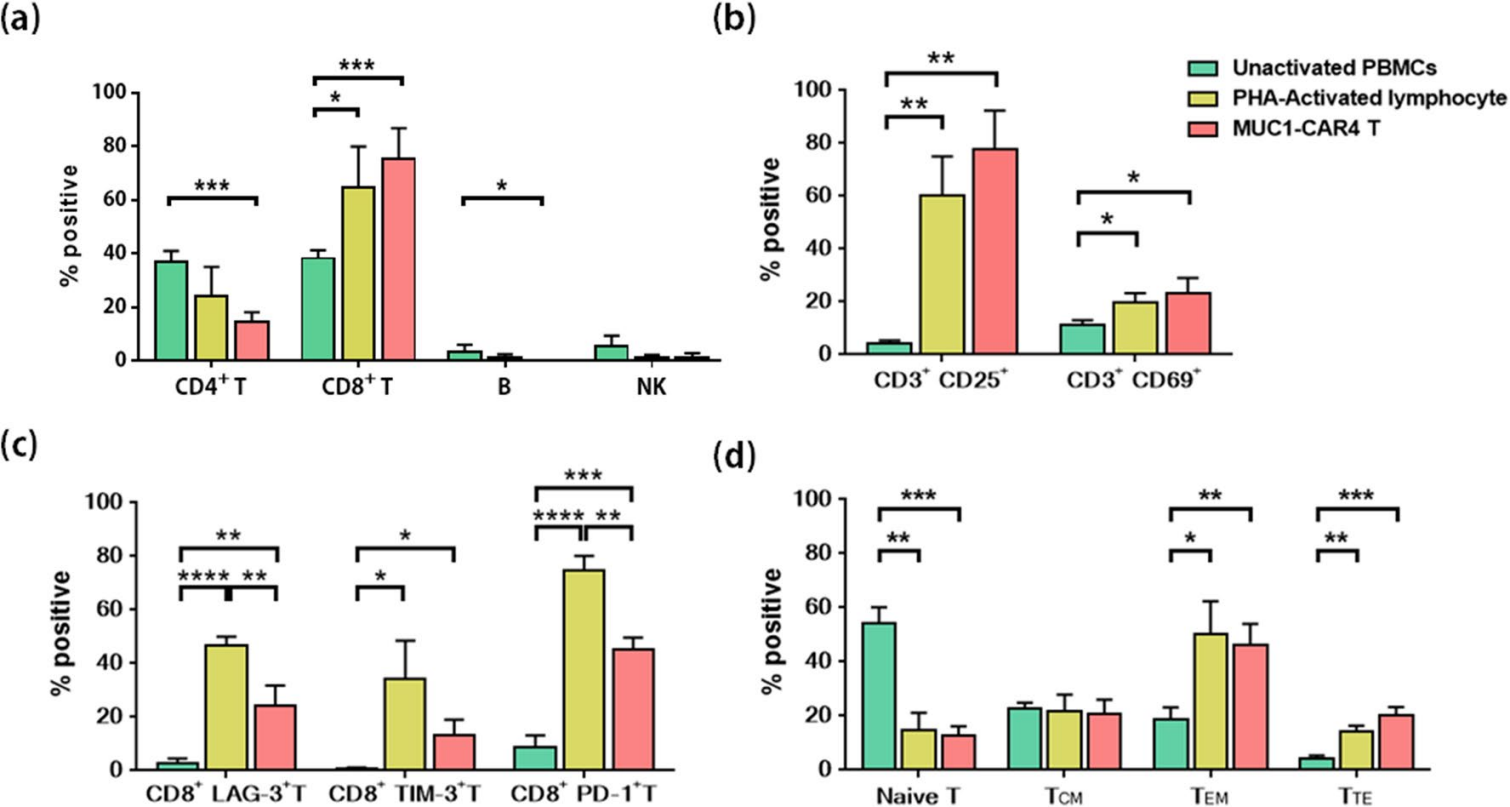
Phenotypes, activation and exhaustion markers, and memory subsets of anti-MUC1-CAR4 T cells analyzed by flow cytometry. The phenotypic analyses of unactivated PBMCs (green), PHA-activated lymphocyte (yellow), and anti-MUC1-CAR4 T cells (red) were summarized: (**a**) cellular phenotypes (n = 4, mean ± SD); (**b**) activation markers (CD25 and CD69) (n = 3, mean ± SD); (**c**) exhaustion markers (LAG-3, TIM-3, and PD-1) (n = 3, mean ± SD); and (**d**) memory subsets (n = 5, mean ± SEM). The statistical differences were analyzed by Student *t*-test (asterisks represent *p*-values: *p < 0.05, **p < 0.01, ***p < 0.001, ****p < 0.0001).

To compare phenotypes of anti-MUC1-CAR4 T cells with other cells, cells were collected in three periods, including before activation (unactivated PBMCs), after activation (PHA-activated lymphocytes), and after transduction (anti-MUC1-CAR4 T cells), and then they were analyzed by flow cytometry. Cytotoxic T (CD3^+^ CD8^+^) cells were significantly increased in PHA-activated lymphocytes (*p*-value 0.0133) and in anti-MUC1-CAR4 T cells (*p*-value 0.0009) compared to unactivated PBMCs. In contrast, helper T (CD3^+^ CD4^+^), B (CD3^-^ CD19^+^), and NK (CD3^-^ CD16^+^ CD56^+^) cells were decreased after PHA activation (Fig. 4a). The representative data and gating strategy of the cell phenotypes are shown in Supplementary Fig. 4c. The results showed that CD8^+^ T cells were prominent after the activation and transduction.

Compared to unactivated PBMCs, the PHA-activated cells and anti-MUC1-CAR4 T cells showed significantly increased activation markers both CD3^+^ CD25^+^ (*p*-value 0.0027 and 0.0011, respectively) and CD3^+^ CD69^+^ (*p*-value 0.0169 and 0.0231, respectively) (Fig. 4b). Furthermore, the PHA-activated and anti-MUC1-CAR4 T cells expressed exhaustion markers as demonstrated in Fig. 4c. Interestingly, the anti-MUC1-CAR4 T cells exhibited lower exhaustion markers, including CD8^+^ LAG-3^+^ T cells (*p*-value 0.0091), CD8^+^ TIM-3^+^ T cells (*p*-value 0.0820) and CD8^+^ PD-1^+^ T cells (*p*-value 0.0021), when they were compared to the PHA-activated lymphocytes. The gating strategies of activation and exhaustion markers were shown in Supplementary Fig. 4d.

T cell phenotypes and memory subsets, including naïve T cells (CD3^+^ CD45RA^+^ CD62L^+^), terminal effector T cells; T_TE_ (CD3^+^ CD45RA^+^ CD62L^-^), effector memory T cells; T_EM_ (CD3^+^ CD45RA^-^ CD62L^-^), and central memory T cells; T_CM_ (CD3^+^ CD45RA^-^ CD62L^+^) were investigated. Compared to unactivated cells, the activated lymphocytes and anti-MUC1-CAR4 T cells showed decreased naïve T cells (from 54.1 ± 15.06% to 14.56 ± 16.28% and 12.57 ± 8.708%; *p*-values 0.0014 and 0.0002, respectively), increased T_TE_ (from 4.18 ± 2.847% to 14.08 ± 5.93% and 20.38 ± 7.18%; *p*-values 0.0318 and 0.0097, respectively), and increased T_EM_ (from 18.96 ± 10.38% to 50.45 ± 29.4% and 46.48 ± 18.42%; *p*-values 0.0042 and 0.0004, respectively). Of note, the memory T cell subsets of PHA-activated lymphocytes and anti-MUC1-CAR4 T cells were not significantly different (Fig. 4d). The representative raw data of memory T cell subsets is shown in Supplementary Fig. 4b. In summary, the generated anti-MUC1-CAR4 T cells were mainly cytotoxic CD8^+^ T cells with effector memory subset (T_EM_).

### Anti-MUC1-CAR4 T cells increased cytokine production in response to exposure to MUC1-expressing CCA cells

**Figure 5:**
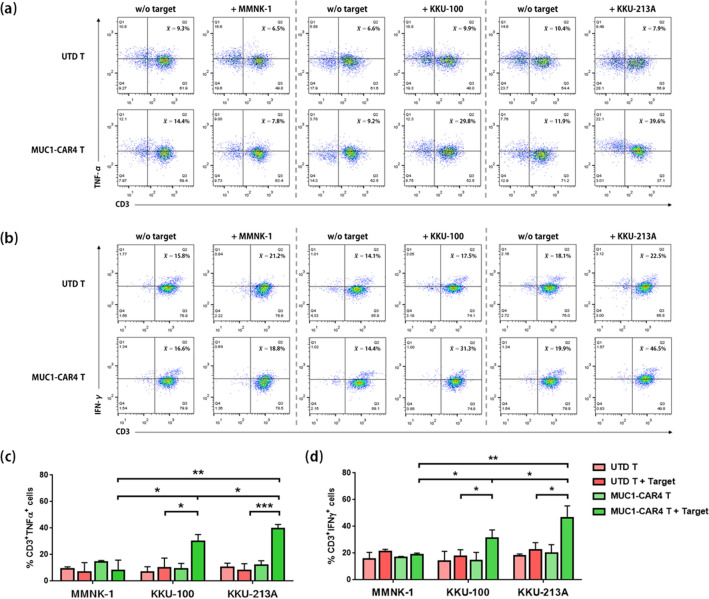
TNF-α and IFN-γ production by anti-MUC1-CAR4 T cells following exposure to CCA cells. The representative cell gating data of (**a**) TNF-α and (**b**) IFN-γ cytokine production in untransduced (UTD) T cells, UTD T cells plus target cells, anti-MUC1-CAR4 T cells alone, and anti-MUC1-CAR4 T cells co-cultured with MMNK-1, KKU100, and KKU-213A. The percentages of TNF-α (**c**) and IFN-γ (**d**) producing cells plotted as bar graphs were individually averaged from 3 independent experiments (mean ± SD). All data were analyzed by Student *t*-test (asterisks represent *p*-values: *p < 0.05, **p < 0.01, ***p < 0.001).

To investigate cytokine production in response to MUC1-expressing cancer cells, MMNK-1, KKU-100, and KKU-213A cells were co-cultured with anti-MUC1-CAR4 T cells at an E:T ratio of 5:1. After six hours of co-culture, the anti-MUC1-CAR4 T cells and UTD control T cells were evaluated for TNF-α (CD3^+^ TNF-α^+^) and IFN-γ (CD3^+^ IFN-γ^+^) production by intracellular staining and flow cytometry. The TNF-α cytokine production was significantly increased in the anti-MUC1-CAR4 T cells exposed to KKU-100 (28.57 ± 5.18%, *p*-value 0.0233) and KKU-213A (39.6 ± 3.12%, *p*-value 0.0008) compared to UTD T cells (9.96 ± 7.38% and 7.98 ± 5.15%, respectively) (Fig. 5a,c). Moreover, the TNF-α production was significantly higher in the anti-MUC1-CAR4 T cells co-cultured with KKU-213A, compared to that with KKU-100 (*p*-value 0.0342). Increased production of IFN-γ by the anti-MUC1-CAR4 T cells was also noted after exposure to KKU-100 (31.33 ± 6.02%, *p*-value 0.0123) and KKU-213A cells (46.5 ± 8.822%, *p*-value 0.0157), compared to UTD T cells (Fig. 5b,d). The significantly higher IFN-γ production in the anti-MUC1-CAR4 T cells exposed to KKU-213A was found when compared with the anti-MUC1-CAR4 T cells exposed to KKU-100 (*p*-value 0.0412). Both TNF-α and IFN-γ cytokines were produced at lower levels when anti-MUC1-CAR4 T cells were exposed to MMNK-1 cells (7.75 ± 8.05% and 18.8 ± 1.21%, respectively), which were not significant difference from those of UTD T cells (9.3 ± 1.43% and 21.2 ± 1.69%, respectively) (Fig. 5). In addition, the significantly higher production of TNF-α and IFN-γ were found in CD8^+^ fraction of the anti-MUC1-CAR4 T cells after co-culture with MUC1-expressing cancer cells (Supplement Fig. 5a,b). In summary, the levels of TNF-α and IFN-γ produced by anti-MUC1-CAR4 T cells were related to MUC1 expression on the cancer cell membrane.

### Cytotoxic function of anti-MUC1-CAR4 T cells on MUC1-expressing CCA cells

**Figure 6:**
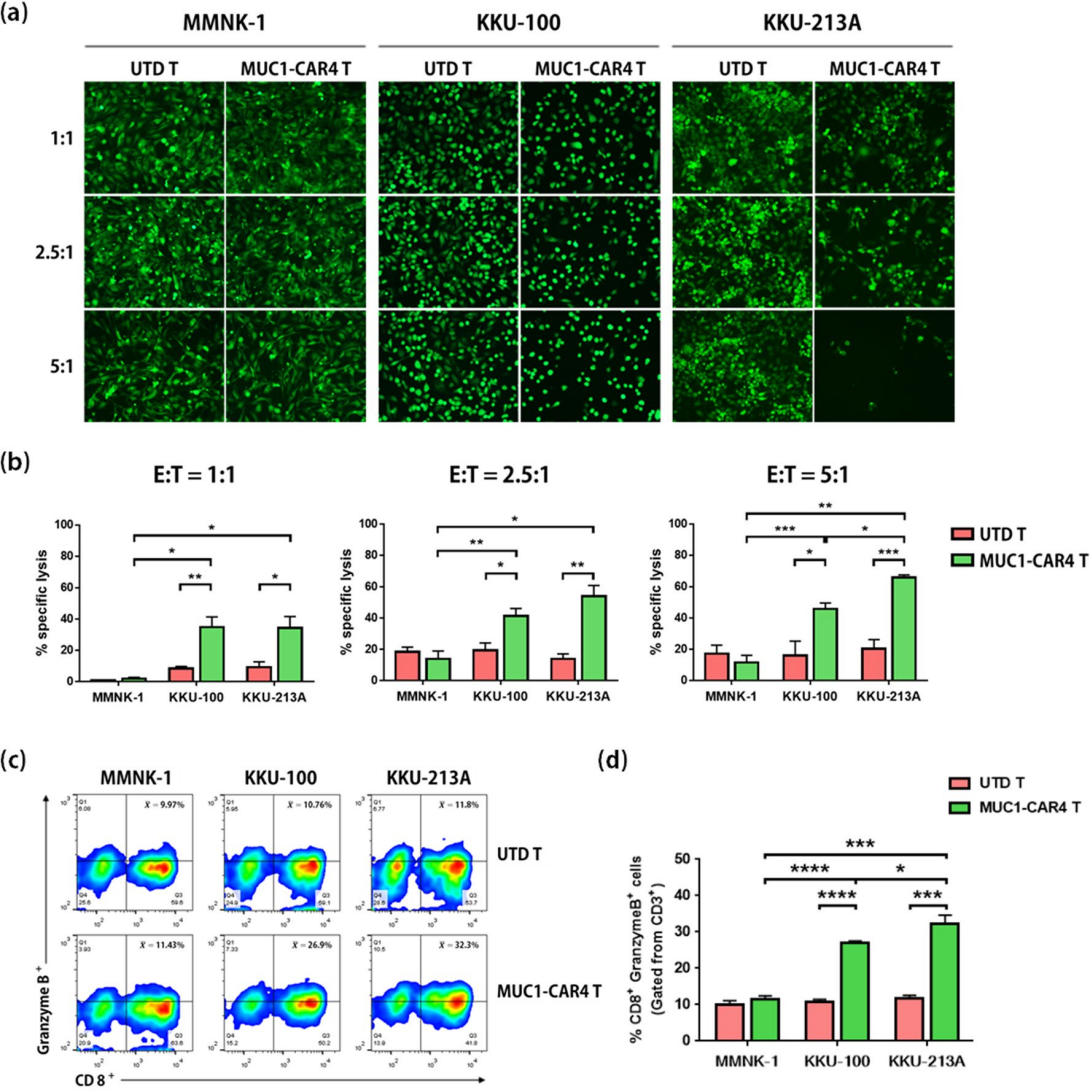
Cytotoxic function of anti-MUC1-CAR4 T cells on MUC1-expressing CCA cells. (**a**) The remaining target cells detected by fluorescence microscopic method after co-culturing with anti-MUC1-CAR4 T cells. (**b**) Specific cell lyses of MMNK-1, KKU-100, and KKU-213A cells after co-culture with the anti-MUC1-CAR4 T cells, examined by luciferase assay at E:T ratios of 1:1, 2.5:1, and 5:1, respectively. Four independent experiments were conducted and summarized (mean ± SEM). (**c**) The representative cell gating to detect granzyme B in UTD T and anti-MUC1-CAR4 T cells. (**d**) The bar graphs showed percentages of the granzyme B-producing cells after exposure to MMNK-1, KKU-100, and KKU-213A. The data were summarized from 3 independent experiments (mean ± SD). The statistical differences were analyzed by Student *t*-test (asterisks represent p-values: *p < 0.05, **p < 0.01, ***p < 0.001).

To examine the cytotoxic function of anti-MUC1-CAR4 T cells, the CAR4 T cells were co-cultured with MMNK-1, KKU-100, and KKU-213A cells, which showed different cell surface expression of MUC1: 4.02 ± 0.994%, 15.14 ± 1.526%, and 58.8 ± 12.59%, respectively (Fig. 2d,e). These non-transformed cholangiocytes (MMNK-1) and CCA cancer cells (KKU-100 and KKU-213A) were genetically engineered to express green fluorescent protein and luciferase. The anti-MUC1-CAR4 T cells were co-cultured with these target cells at E:T ratios of 1:1, 2.5:1, and 5:1 for 18 h. After exposure to the anti-MUC1-CAR4 T cells, KKU-100 and KKU-213A cells were lysed in a dose-dependent manner, but MMNK-1 cells showed low cytolysis (Fig. 6a).

The cytotoxic functions of anti-MUC1-CAR4 T cells on MMNK-1, KKU-100, and KKU-213A cells were also examined by luciferase assay. Compared to UTD T cells, the anti-MUC1-CAR4 T cells showed a cytotoxic effect on KKU-100 and KKU-213A, but not on MMNK-1 cells. The cytotoxic effects of the anti-MUC1-CAR4 T cells on KKU-100 and KKU-213A cells at an E:T ratio of 1:1 were 34.82 ± 13.18% (*p*-value 0.0078) and 34.34 ± 14.62% (*p*-value 0.0213), respectively. At E:T ratios of 2.5:1 and 5:1, the percentages of KKU-100 lysis were slightly increased (41.49 ± 9.38%, *p*-value 0.0160, and 45.88 ± 7.45%, *p*-value 0.0237, respectively). The percentage of KKU-213A lysis after exposure to the anti-MUC1-CAR4 T cells at E:T ratios of 2.5:1 and 5:1 was increased in a dose-dependent manner (54.16 ± 13.56%, *p*-value 0.0018, and 66.03 ± 3.14%, *p*-value 0.0003, respectively). In contrast, the anti-MUC1-CAR4 T cells showed low cytotoxic effect on MMNK-1 cells (1.87 ± 1.75%, 13.96 ± 9.99%, and 11.6 ± 9.21% at E:T ratios of 1:1, 2.5:1, and 5:1, respectively) (Fig. 6b). The cytotoxic function of the anti-MUC1-CAR4 T cells on MMNK-1 and KKU-213A cells after co-culture for 24 h was examined by crystal violet staining method. In correlation with fluorescence detection and luciferase assay (Fig. 6a,b), the anti-MUC1-CAR4 T cells showed cytotoxic effect on KKU-213A, but had no effect on MMNK-1 cells (Supplementary Fig. 5c).

Cytotoxic granzyme B production of the anti-MUC1-CAR4 T cells was measured after co-culture with MMNK-1, KKU-100, and KKU-213A for 6 h. The anti-MUC1-CAR4 T cells increased granzyme B production in an antigen-specific manner after exposure to MUC1-expressing CCA cells, KKU-100 and KKU-213A (26.9 ± 0.61% and 32.3 ± 2.34%, respectively), compared to those of UTD T cells (10.77 ± 0.65% and 11.8 ± 0.79%, respectively). Moreover, the percentage of granzyme B production in the anti-MUC1-CAR4 T cells exposed to KKU-213A cells was significantly higher than that of the anti-MUC1-CAR4 T cells exposed to KKU-100 cells (*p*-value 0.0181). The granzyme B production was not increased when the anti-MUC1-CAR4 T cells were co-cultured with MMNK-1 cells (Fig. 6c,d).

### Anti-MUC1-CAR4 T cells disrupted MUC1-expressing spheroids

**Figure 7:**
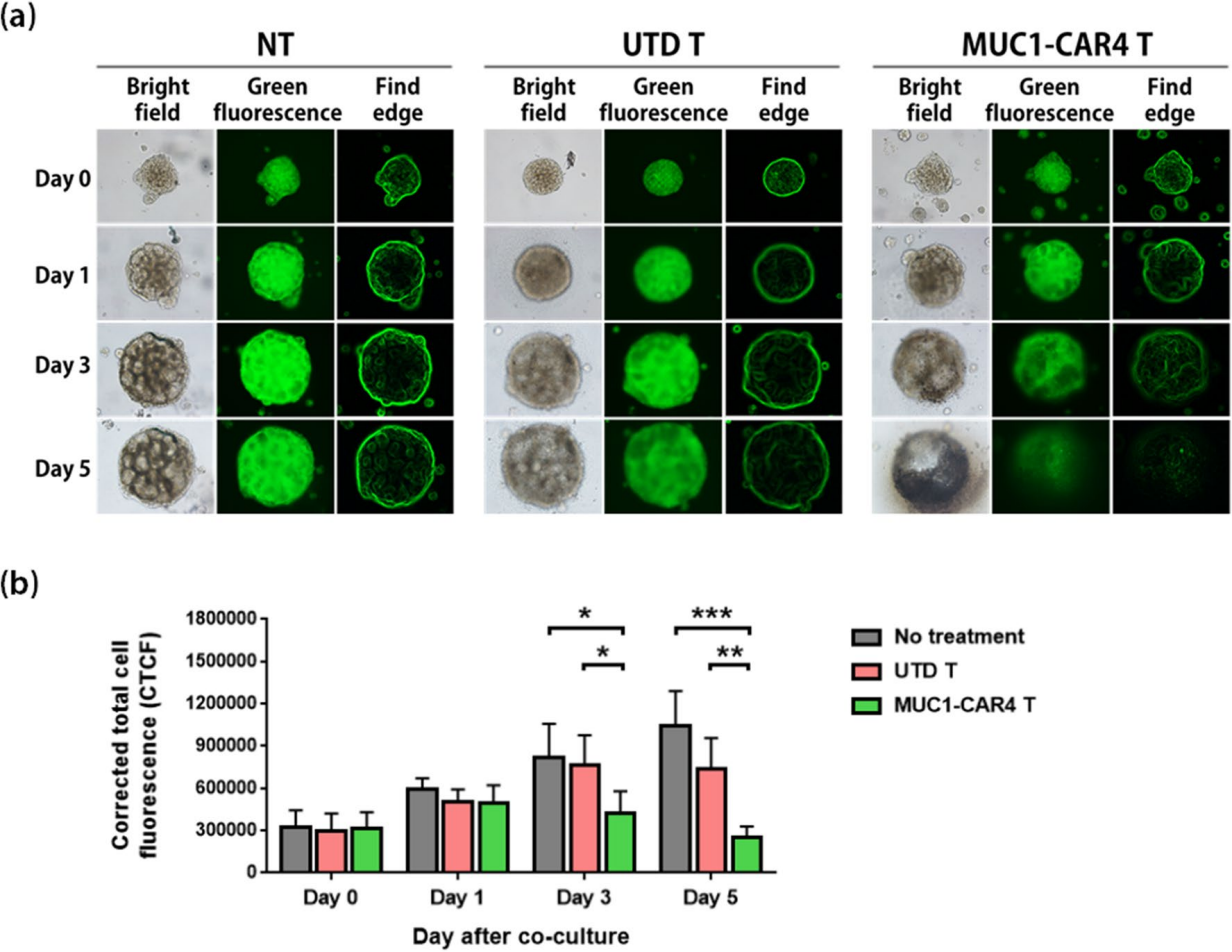
Anti-tumor effect of anti-MUC1-CAR4 T cells against MUC1-expressing KKU-213A spheroids. (**a**) Representative results of KKU-213A spheroids expressing a green fluorescence protein; left panel: no treatment (NT); middle panel: spheroids co-cultured with untransduced T cells (UTD T); right panel: spheroids co-cultured with anti-MUC1-CAR4 T cells (E:T ratio of 10:1). The spheroids were captured under a fluorescence microscope at 10 × objective lens. (**b**) The bar graphs represent summarized data of corrected total cell fluorescence (CTCF) from 4 independent donors (mean ± SD). The statistical differences were analyzed by Student *t*-test (asterisks indicate *p*-values: *p < 0.05, **p < 0.01, ***p < 0.001).

To examine the ability of anti-MUC1-CAR4 T cells to disrupt MUC1-expressing KKU-213A spheroids, UTD T cells or anti-MUC1-CAR4 T cells were co-cultured with the KKU-213A spheroids at an E:T ratio of 10:1. After co-culturing, the disruptions of KKU-213A spheroids on days 3 and 5 were evidenced by the reduction of green fluorescence signals, losing of spheroid structures, and disrupted spheroid edges (Fig. 7a). The KKU-213A spheroids without anti-MUC1-CAR4 T cells (no treatment; NT) were used as a negative control, showing the intact spheroid structures and edges. Significant reductions of green fluorescent signals of viable KKU-213A spheroids treated with the anti-MUC1-CAR4 T cells were compared to that of no treatment condition (NT) or UTD T cells on day 3 (*p*-value 0.0315 and *p*-value 0.0373, respectively) and on day 5 (*p*-value 0.0008 and *p*-value 0.0057, respectively) (Fig. 7b). Thus, the anti-MUC1-CAR4 T cells showed effective anti-tumor responses against the MUC1-expressing CCA spheroids.

## Discussion

Cholangiocarcinoma (CCA) is a bile duct epithelial malignancy that causes high mortality worldwide, and it has a high prevalence in the Northeastern population of Thailand^27^. Current treatments, including surgery, radiation, chemotherapy, targeted therapy, and liver transplantation, are not effective in advanced and metastatic CCA, resulting in high recurrence rate and low long-term survival^28,29^. Treatment of CCA patients with immunotherapy showed improvement in therapeutic outcome^1^. However, some limitations were also observed. The anti-cancer functions of DC-based and also T cell-infiltrating lymphocytes (TILs) therapy could be obstructed by downregulation of MHC molecule, and upregulation of immune checkpoint ligand on cancer cells, which are characterized as immune escape mechanisms^30,31^. In the present study, we aimed to develop adoptive T cell therapy using CAR T cells, a highly effective modality for cancer treatment, and to investigate their cytotoxic function against CCA. CAR T cells recognize a specific TAA on cancer cells. Thus, selection of an appropriate antigen with properties that are well characterized and that are known to affect T cell response with low or no adverse side effects is essential for generation of CAR T cells. Mucin 1 (MUC1) was the specific TAA that was selected for CAR T cell targeting in the present study since it was reported as a second most attractive target antigen for cancer immunotherapy^32^.

We confirmed that MUC1 is a potential cancer antigen for CCA. MUC1 expression was investigated in four CCA tissues by IHC, and it was found that MUC1 was overexpressed both in the cytoplasm and on the membrane of CCA cells (Fig. 1). Expression patterns of MUC1 in CCA tissues were similar to that of previous study^18^, which reported the detection of MUC1 in Thai CCA patients from Khon Kaen Province. The researchers in that previous study observed MUC1 expression in 77% (67/87) of CCA tissues. Strong expression of MUC1 was also reported in 86.5% (32/37) of CCA tissues in Chinese patients^19^, and in 65.8% (56/85) of CCA tissues in Korean patients^20^. The expression of MUC1 was low in adjacent normal bile duct epithelial cells. Similar to a previous report^19^, we could not detect MUC1 expression in adjacent normal hepatocytes (Fig. 1b). However, MUC1 was found to express in hepatocellular carcinoma tissues^33^. Therefore, these results suggested MUC1 as a potential target antigen for immunotherapy using CAR T cells.

Importantly, there has been no report of MUC1 expression in CCA cell lines. We, therefore, decided to investigate MUC1 expression in non-cancer and CCA cell lines, including MMNK-1, KKU-055, KKU-100, and KKU-213A, compared to MUC1 expression in breast cancer MCF-7 cells, which were previously reported to have high expression (Fig. 2)^26^. MUC1 was highly expressed both in the cytoplasm and on cell surface of KKU-213A cells, while intermediate cell surface expression was found in KKU-055 and KKU-100 cells. In contrast, MUC1 was weakly expressed on the cell surface of MMNK-1 cells (Fig. 2c). Thus, these cell lines with different levels of MUC1 expression were valuable for testing cytotoxicity by CAR T cells.

Specific co-stimulatory molecules are known to regulate anti-tumor function, proliferation, cytokine production, and survival of CAR T cells. In the present study, we generated lentiviral pCDH vector containing scFv derived from the reported antibody specific to MUC1^11^ that was linked to CAR cassette containing CD28, CD137, CD27, and CD3ζ (anti-MUC1-CAR4) (Fig. 3a). CD28 is expressed both in resting and activated T cells. It was reported to promote T cell proliferation, IL-2 and Th1 cytokine production, and activation-induced cell death (AICD) resistance of CAR T cells^34^. CAR T cells containing the CD28 molecule could improve anti-cancer functions and persistence both in vitro and in vivo compared to first-generation CAR and 4-1BB-based CAR T cells^35^. Moreover, it was shown to enhance CAR T cell activity, expansion, and longer persistence in lymphoma patients^36^. CD137 (or 4-1BB) is expressed on resting CD8^+^ T cells and upregulated on both activated CD4^+^ and CD8^+^ T cells. CD137 activation enhances IL-2 and IFN-γ production in CD8^+^ T cells, while it induces IL-2 and IL-4 production in CD4^+^ T cells. It preferentially promotes CD8^+^ T cell proliferation^37^, granzyme B expression, IFN-γ and TNF-α production, and apoptotic resistance in vitro^38^. Finally, CD27 incorporation was expected to enhance apoptotic resistance of CAR T cells. CD27-based CAR T cells showed enhancement of the cell persistence compared to CD28-based CAR T cells^39^. Therefore, incorporation of these 3 co-stimulatory molecules could promote anti-tumor activities, proliferation, and survival of our CAR T cells. Expression of our anti-MUC1-CAR molecules was confirmed in HEK293T cells (Supplementary Fig. 3a,b) and lentivirus transduced T cells (Fig. 3b,c), at high transduction efficiency. We compared killing activities of anti-MUC1-CAR2 T cells and that of anti-MUC1-CAR4 T cells and found that the latter had significantly higher killing activities than the former in the testing against MUC1-expressing CCA, KKU-213A cells (Fig. 3d). Proliferation of the anti-MUC1-CAR4 T cells was also higher than that of the anti-MUC1-CAR2 T cells after co-culture with MUC1-expressed KKU-213A cells (Fig. 3e). The higher killing activities and cellular expansion abilities of the anti-MUC1-CAR4 T cells might be attributable to the presences of CD28^34,35^ and CD27^39^ co-stimulatory molecules that were reported to promote T cytotoxic activities and T cell expansion. Phytohemagglutinin (PHA) was used to activate lymphocytes in our CAR T cell production for the in vitro experimental studies; this might not be suitable for clinical applicability. In the CAR T cell production for clinical studies, CD3/CD28 monoclonal antibodies should be used instead of PHA to activate lymphocytes.

The phenotypes of cells showed increased cytotoxic T (CD3^+^ CD8^+^) cells (Fig. 4a). Expansion of CD8^+^ T cells might be caused by IL-2, IL-7, and IL-15 cytokines in culture condition. Another study showed that significant expansion of antigen-specific CD8^+^ T cells was observed after antigen stimulation and addition of IL-2 or IL-15 cytokine^40^. The PHA used in cell activation step was also reported to promote CD8^+^ T cell expansion^41^. Furthermore, CD27 and CD137 molecules in the CAR4 T cells preferentially supported CD8^+^ T cell proliferation^37^. The anti-MUC1-CAR4 T cells also showed decreased exhaustion markers compared to PHA-activated lymphocytes (Fig. 4c). Our result was agreeable with the previous study that reported decreased exhaustion markers in CAR T cell containing CD137 molecules^42^. The major phenotype of the anti-MUC1-CAR4 T cells was effector memory (T_EM_) T cell phenotypes (Fig. 4d), which may be an effect of IL-2, IL-7 and IL-15 cytokines in the culture condition. These cytokines were reported to be able to enrich of memory T cells^43,44^. The T_EM_ can exhibit the highest level of cytotoxicity of all the memory cell subtypes^44^. Similar results were also reported in previous studies^45,46^.

We investigated cytokine production of anti-MUC1-CAR4 T cells in response to exposure to MMNK-1, KKU-100, and KKU-213A. Here we found that intracellular TNF-α and IFN-γ cytokines were only produced by the anti-MUC1-CAR4 T cells after co-culture with MUC1-expressing CCA cells (Fig. 5), suggesting their antigen-specific responses. TNF-α is an inflammatory cytokine that is produced by macrophage and activated T cells, contributing to target cell necrosis^47^. IFN-γ cytokine is mainly secreted from T and NK cells and contributes importantly to cancer cell clearance^48^. Our results demonstrated that cytotoxic T cells were the major cell population producing TNF-α and IFN-γ cytokines (Supplementary Fig. 5a,b). The enhanced TNF-α and IFN-γ productions were also reported in other CAR T cell studies^14,22,23^.

Co-culturing of anti-MUC1-CAR4 T cells with CCA cells showed significant cancer cell lysis, which correlated with cell surface expression of MUC1; however, this effect was not observed with MMNK-1 cells (Fig. 6,b). In addition, the killing efficiency of anti-MUC1-CAR4 T cells against CCA cells was corresponded with the levels of granzyme B production, which depended on MUC1 expression levels on CCA cell surface (Figs. 2d,e, 6c,d). In general, granzyme B is a pro-apoptotic protein produced by effector T cells that plays important role in target cell apoptosis^49^. Our data suggested that TNF-α, IFN-γ, and granzyme B produced by anti-MUC1-CAR4 T cells contribute to CCA cytolysis. Although the general mechanisms of cancer cytolysis by CAR T cells have already been established^49^, further studies using specific antibody blocking could be conducted to confirm the roles of these cytokines in cancer cell killing. Our anti-MUC1-CAR4 T cells showed potent anti-cancer effect comparable to that of other studies in different cancer models using second- and third-generation anti-MUC1-CAR T cells^11,22,23^, and also inverted cytokine receptor (IL-4R/IL-7) CAR T cells^12^. Therefore, the results of our study support the therapeutic potential of anti-MUC1-CAR4 T cells for further development into an alternative CCA treatment. Interestingly, we have also investigated the killing efficacy of the anti-MUC1-CAR4 T cells by using three-dimensional (3D) spheroid culture of KKU-213A cells. The 3D spheroids contain characteristics and morphology closer to solid tumors; therefore, the assay using 3D spheroid will be more illustrative than 2D-cell-based assay^50^. Recently, our group has reported the use of 3D spheroids to evaluate cytotoxic effects of CAR T cells in CCA and breast cancer^14,15^. In the present study, our results showed that the KKU-213A spheroids were disrupted by the anti-MUC1-CAR4 T cells (Fig. 7), strongly suggesting the potential of the adoptive T cell therapy using anti-MUC1-CAR4 T cells in CCA. The anti-MUC1-CAR4 T cell proliferation was evaluated up to 7 days in the 2D co-culture (Fig. 3e) and the anti-tumor effect of the anti-MUC1-CAR4 T cells could be observed up to 5 days in 3D spheroid co-culture. These may partially suggest their persistence in the co-culture conditions. However, the long-term persistence of the anti-MUC1-CAR4 T cells and their anti-tumor activities should be further investigated.

Cancer heterogeneity, tumor microenvironment, assessment of anti-tumor efficacy of CAR T cells, and safety are major concerns in the development of CAR T cell-based therapies. MUC1 expression was detected in CCA patient tissues from many countries, but the expression varied, and the highest MUC1 expression was 86.5%. Therefore—some, but not all, CCA patients could be treated with this therapy. MUC1 expression should be firstly examined in patient tissues before treatment selection. For patients showing low MUC1 expression, combined treatments, including dual-CAR T cells or combination of anti-MUC1-CAR T cells with CAR T cells targeting other CCA-associated antigens, should be further investigated. Since the previous evidence showed that gemcitabine, a chemotherapeutic agent, induced MUC1 upregulation in primary intrahepatic cholangiocarcinoma cells from patients^51^, the combination of anti-MUC1-CAR T cells with gemcitabine may be a potential treatment in patients with CCA. Elements within the tumor microenvironment, such as immune checkpoint molecules (PD-1/PD-L1) and immunosuppressive cytokines, should also be addressed. To overcome these problems, the CAR T cells should be able to disrupt or block the immune checkpoint PD-1/PD-L1, modify to express switch receptor (PD-1-CD28), or modify by addition of inverted cytokine receptor (IL-4R/IL-7)^52^ to improve the CAR T cell functions within the tumor microenvironment. To address the safety issue, the CAR T cells can be modified to express suicide genes, including herpes simplex virus thymidine kinase (HSV-tk), inducible caspase 9 (iCasp9), and truncated epithelial growth factor receptor (tEGFR), which can enable CAR T cell elimination^53^. At present, there are 11 active phase I/II clinical trials of anti-MUC1-CAR T cells against multiple cancer models, some of which are combined therapy with checkpoint blockade^25^. Finally, our anti-MUC1-CAR4 T cells and their safety for use in a CCA treatment setting require further investigation in an animal model and clinical trials.

In conclusion, we have engineered the anti-MUC1-CAR4 and used lentiviral vector delivery to express this anti-MUC1-CAR4 in human T cells, which were mainly of the CD8^+^ T cell subset with effector memory phenotypes and low expression of exhaustion markers. These anti-MUC1-CAR4 T cells showed anti-cancer activity against MUC1-expressing CCA cells by increased production of anti-tumor cytokines (TNF-α and IFN-γ), pro-apoptotic protein (granzyme B), and induction of CCA cell lysis both in 2D and 3D (spheroid) co-cultures. This is the first study showing the potential of anti-MUC1-CAR4 T cells in CCA. The results of this study are beneficial for further development of CAR T cell therapy against CCA.

## Materials and methods

### Ethical approval statement

The guidelines and uses of human blood and tissue materials for research were approved by Institutional Ethical Committees. For the use of blood samples from healthy donors, the protocol and written informed consent documents were approved by the Siriraj Institutional Review Board (number *Si* 101/2020). The use of CCA tissue samples was approved by the Ethics Committee for Human Research, Khon Kaen University (no. HE591063) and the experiments were conducted at Khon Kaen University. All the methods were performed in accordance with the approved guidelines.

### Cell lines and culture condition

MMNK-1 (cholangiocytes), KKU-055, and KKU-213A (cholangiocarcinoma), and MCF-7 (breast cancer) cell lines were cultured in Dulbecco's Modified Eagle Medium (DMEM), while KKU-100 cells were maintained in DMEM-F12 media containing 10% heat-inactivated fetal bovine serum (FBS) and 100 μg/ml of streptomycin and penicillin. Peripheral blood mononuclear cells (PBMCs) from healthy donors were cultured in Roswell Park Memorial Institute (RPMI)-1640 medium supplemented with 5% heat-inactivated human AB serum (R5). Activated PBMCs were cultured in R5 containing recombinant human (rh) IL-2, IL-7, and IL-15 cytokines.

### Immunohistochemistry (IHC)

Tissues were de-paraffinized and rehydrated with stepwise decreasing concentrations of ethanol. Subsequently, antigen retrieval was performed using sodium citrate buffer (pH 6.0) with 0.05% Tween-20 and microwave oven. After adding 0.3% hydrogen peroxide, the tissues were blocked with 5% skim milk and stained with anti-MUC1 antibody (VU4H5, Santa Cruz Biotechnology, Inc., Dallas, TX, USA). HRP-conjugated EnVision secondary antibody (Dako, CA, USA) was added followed by 3, 3′-diaminobenzidine (DAB) for signal development. The tissues were then counterstained with hematoxylin, mounted, and visualized under a light microscope.

### Construction of lentiviral encoded anti-MUC1-CARs

To generate a lentiviral plasmid (pCDH) construct encoding for an anti-MUC1-CAR4, an anti-MUC1 scFv containing a [G4S]_3_ linker derived from the HMFG2 monoclonal antibody (a gift of Dr. John Maher, King’s College London)^11^ was codon optimized. The synthesized cDNA (Integrated DNA Technologies, Coralville, IA, USA) was inserted between an upstream EF-1α and CD8α leader sequence and downstream CD8 hinge and transmembrane domain. The CAR endodomain consisted of a tandem fusion of the intracellular domains of CD28, CD137, CD27, and CD3ζ respectively. Briefly, the DNA fragment was amplified using specific primers, digested with *EcoRI* and *MreI* enzymes, ligated with pCDH-CAR vector, transformed into competent *E. coli* cells, screened by colony PCR, and verified by Sanger DNA sequencing (Supplementary Table 1 and Supplementary Fig. 2). Anti-MUC1-CAR2 construct was created by replacement of anti-MUC1-CAR4 in the region of CD28/CD137/CD27/CD3ζ domains with CD137/CD3ζ domains by cleavages with *MreI* and *NotI* enzymes.

To produce lentiviral particles, the anti-MUC1-CAR plasmid constructs were transfected into Lenti-X 293 T cells (Takara Bio Inc., Shiga, Japan) with psPAX2 and pMD2.G plasmids. Supernatants were collected at 48 and 72 h.

### Transfection

The anti-MUC1-CAR2 and anti-MUC1-CAR4 plasmid constructs were transfected into HEK293T cells using Lipofectamine 2000 (Thermo Fisher Scientific, Waltham, MA, USA), following the manufacturer’s protocol. The transfected cells were collected for anti-MUC1-CAR molecule detection by immunoblotting and flow cytometry.

### Peripheral blood mononuclear cell (PBMC) preparation and activation

PBMCs were collected using Corning Lymphocyte Separation Medium (Corning Inc., Corning, New York, USA) following the manufacturer’s process. PBMCs were then plated in culture dish to allow adherence of unwanted monocytes. After 6 h, non-adherent cells were gently collected as a source of lymphocytes and activated using 5 μg/mL of phytohemagglutinin (PHA)-L (Roche, Basel, Switzerland) for three days.

### MUC1-targeting CAR T cell production

The activated lymphocytes were mixed with 10 μg/mL of protamine sulfate (Mochida Pharmaceutical Co., Ltd., Tokyo, Japan) and lentiviral supernatants (added at a multiplicity of infection of 10) and centrifuged for 90 min at 1200×*g* in plates coated with Retronectin (Takara Bio Inc., Shiga, Japan). The transduced lymphocytes were maintained in culture medium containing rhIL-2 (20 ng/mL), rhIL-7 (10 ng/mL), and rhIL-15 (40 ng/mL). On days 5–7 after transduction, anti-MUC1-CAR expression was detected on transduced cell surface by flow cytometry as indicated below. Activated lymphocytes mixed with protamine sulfate that were not transduced with lentiviral supernatants were included as untransduced control cells.

### Immunoblot analysis

MMNK-1, KKU-055, KKU-100, KKU-213A, and MCF-7 cells were collected and lysed using 8 M urea lysis buffer. Total protein lysates were run through SDS-PAGE. MUC1 protein expression was detected using anti-MUC1 antibody (VU4H5). Band intensity was determined using ImageJ software^54^ and normalized with β-actin. CAR expression in transfected HEK293T cells was detected using anti-CD3ζ (Santa Cruz Biotechnology, Inc., Dallas, TX, USA).

### Immunofluorescence assay (IFA)

The studied CCA cells were plated on coverslips, fixed with 4% paraformaldehyde, blocked with 1% BSA, and stained with anti-MUC1 antibody (EP1024Y; Abcam, Cambridge, UK). MUC1 expression imaging was performed on a Zeiss LSM 800 confocal microscope (Zeiss, Jena, Germany).

To test cytolytic activity, stably wasabi-luciferase-expressing KKU-213A cells were co-cultured with anti-MUC1-CAR T cells at effector to target (E:T) ratios of 5:1, 2.5:1, and 1:1. After 18 h of co-culture, the CAR T cells were removed and the remaining target cells were captured under fluorescence microscope.

### Proliferation assay

The anti-MUC1-CAR2 T cells, anti-MUC1-CAR4 T cells, and untransduced (UTD) T cells were co-cultured with immortal cholangiocytes (MMNK-1) or MUC1-expressing CCA (KKU-213A cells) at E:T ratio of 1:10 for 3 and 7 days. The total cell numbers of the CAR T cells collected from co-cultured supernatant were counted by Countess II FL Automated Cell Counter (Thermo Fisher Scientific, Waltham, MA, USA). The total cell numbers were normalized with seeding cells (Day 0) and plotted as a fold-change bar graph.

### Flow cytometry

The studied cells were freshly stained with anti-MUC1 antibody (EP1024Y) diluted to 1:50 in 2% FBS in PBS. Antibodies that were used to investigate the cell phenotypes including anti-CD3-FITC, CD4-APC, CD8-APC, CD19-APC, CD16-APC, CD69-APC, and CD62L-APC were purchased from ImmunoTools GmbH (Friesoythe, Germany), anti-CD45RA-PE-cyanine7 and PD-1-PE from Invitrogen (Carlsbad, USA), anti-CD56-PE, LAG-3-PE and TIM-3-PE from BioLegend (San Diego, USA), and anti-CD25-PerCP-cyanine5.5 from eBioscience, Inc. (California, USA).

To detect the CARs expression on transduced lymphocytes, the transduced cells were harvested, blocked with 1% BSA, and stained with biotin-conjugated protein-L (Thermo Fisher Scientific, Waltham, MA, USA). Alexa Fluor 488 dye-conjugated streptavidin was finally added. The data were acquired on a BD FACSVerse or BD Accuri C6 flow cytometer (BD Biosciences, Franklin Lakes, NJ, USA) and analyzed with FlowJo software version 10.

### Intracellular cytokine production

Anti-MUC1-CAR4 T cells were co-cultured with MMNK-1, KKU-100, and KKU213A cells at an E:T ratio of 5:1. Brefeldin A was added after one hour of co-culture and further incubated for 5 h. The CAR4 T cells were harvested, stained with anti-CD3-FITC and anti-CD8-APC antibodies, and fixed with 1% formaldehyde. The cells were permeabilized and stained with anti-TNF-α or anti-IFN-γ-PE antibody (ImmunoTools GmbH, Friesoythe, Germany) diluted in 0.1% saponin. The TNF-α and IFN-γ cytokine production in the anti-MUC1-CAR4 T cells was measured by flow cytometry.

### Luciferase assay

Anti-MUC1-CAR2 T and anti-MUC1-CAR4 T cells were co-cultured with stably wasabi-luciferase-expressing target cells at E:T ratios of 1:1, 2.5:1, and 5:1. After 18 h, the CAR T cells were removed and the remaining target cells were detected for luciferase activity using a Pierce Firefly Luciferase Glow Assay Kit (Thermo Fisher Scientific, Waltham, MA, USA). Luminescence was immediately determined using a luminometer. The percentage of killing was calculated using this formula [% specific killing = 100 − ((luciferase activity from well with effector and target cells)/(luciferase activity from well with target cells) × 100)].

### Granzyme B detection

The anti-MUC1-CAR4 T cells were co-cultured with the MMNK-1, KKU-100, or KKU-213A cells at E:T ratio of 5:1. After 1 h, Brefeldin A (1 × dilution) was added into the culture medium and further incubated for 5 h. The anti-MUC1-CAR4 T cells were collected and stained with anti-CD3-PerCP and anti-CD8-APC antibodies (ImmunoTools GmbH, Friesoythe, Germany). After fixation with 1% formaldehyde for 10 min, the anti-MUC1-CAR4 T cells were permeabilized and stained with anti-granzyme B-FITC (ImmunoTools GmbH) in 0.1% saponin solution.

### Three-dimension spheroid killing assay

To generate three-dimension (3D) spheroids, stably expressing wasabi-luciferase KKU-213A cells (2 × 10^3^ cells) were mixed on ice with 5 μL of Corning Matrigel Basement Membrane Matrix. The mixture was then plated into Corning Ultra-Low Attachment Surface Products (Corning Inc., NY, USA). The plate was centrifuged at 1000×*g*, 4 °C, for 10 min. After 2 days of 3D spheroid formation, the anti-MUC1-CAR4 T cells were added into the plate at E:T ratio of 10:1. The KKU-213A spheroid morphology was captured at day 0, 1, 3, and 5 after co-culture by a fluorescence microscope (Nikon, Tokyo, Japan). The spheroid edge and corrected total cell fluorescence (CTCF) were analyzed by ImageJ software^54^. The CTCF was calculated by the following formula: CTCF = Integrated Density − (Area of selected cell × Mean fluorescence of background readings).

### Statistical analysis

Data are representative of 3–5 independent experiments. The data were statistically analyzed using GraphPad Prism version 7 (GraphPad Software, Inc., San Diego, CA, USA, www.graphpad.com). The results are shown as mean ± standard deviation (SD) or ± standard error of the mean (SEM). Significant differences between compared data were determined by unpaired *t*-test (*p < 0.05, **p < 0.01, ***p < 0.001, ****p < 0.0001).

## Supplementary Information

Supplementary Information.
