# Supplementary material for: Anti-mucin 1 chimeric antigen receptor T cells for adoptive T cell therapy of cholangiocarcinoma
Source: Sci Rep. 2021 Mar 18;11:6276. doi: 10.1038/s41598-021-85747-9 (PMC7973425; doi:10.1038/s41598-021-85747-9)
Supplement: Supplementary file 1 — Supplementary Information. [file 41598_2021_85747_MOESM1_ESM.pdf]

Supplementary data

Anti-mucin 1 chimeric antigen receptor T cells for adoptive T cell therapy of cholangiocarcinoma

Kamonlapat Supimon[1,2], Thanich Sangsuwannukul[1,2], Jatuporn Sujjitjoon[1,3], Nattaporn Phanthaphol[1,2], Thaweesak Chieochansin[1,3], Naravat Poungvarin[4], Sopit Wongkham[5,6], Mutita Junking[1,3], Pa-thai Yenchitsomanus[1,3*]

[1] *Siriraj Center of Research Excellence for Cancer Immunotherapy (SiCORE-CIT), Research Department, Faculty of Medicine Siriraj Hospital, Mahidol University, Bangkok, Thailand*

[2] *Graduate Program in Immunology, Department of Immunology, Faculty of Medicine Siriraj Hospital, Mahidol University, Bangkok, Thailand*

[3] *Division of Molecular Medicine, Research Department, Faculty of Medicine Siriraj Hospital, Mahidol University, Bangkok, Thailand*

[4] *Department of Clinical Pathology, Faculty of Medicine Siriraj Hospital, Mahidol University, Bangkok, Thailand*

[5] *Department of Biochemistry, and Center for Translational Medicine, Faculty of Medicine, Khon Kaen University, Khon Kaen, Thailand*

[6] *Cholangiocarcinoma Research Institute, Khon Kaen University, Khon Kaen, Thailand*

***Corresponding Author** Professor Pa-thai Yenchitsomanus, Ph.D.

Division of Molecular Medicine, Research Department

Faculty of Medicine Siriraj Hospital, Mahidol University

2 Wanglang Road, Bangkoknoi, Bangkok 10700, Thailand

Tel: (+66) 2-419-2777; Fax: (+66) 2-411-0169

E-mail: pathai.yen@mahidol.edu; ptyench@gmail.com

Supplementary Table 1. Primers for amplification of inserted fragment and colony PCR

Primer name	Nucleotide sequence	Number of nucleotides	Tm (°C)	Product size (bp)
**Primers for amplification of anti-MUC1 scFv sequence (inserted fragment)**				
MUC1-scFv-F (*EcoR*I)	5'-ACGAATTCATGGCTCTCCCAGTGACTGC-3'	28	65	1,698
MUC1-scFv-R (*Mre*I)	5'- TATCCGCCGGCGgTTTACCCGGAGACAGG- 3'	29	69	
**Primers for colony PCR**				
pCDH-F	5'-GAGTTTCCCCACACTGAGTG-3'	20	56	1,867
MUC1-scFv-R (*Mre*I)	5'- TATCCGCCGGCGgTTTACCCGGAGACAGG- 3'	29	69	

Abbreviations: PCR, polymerase chain reaction; Tm, temperature; bp, base pairs

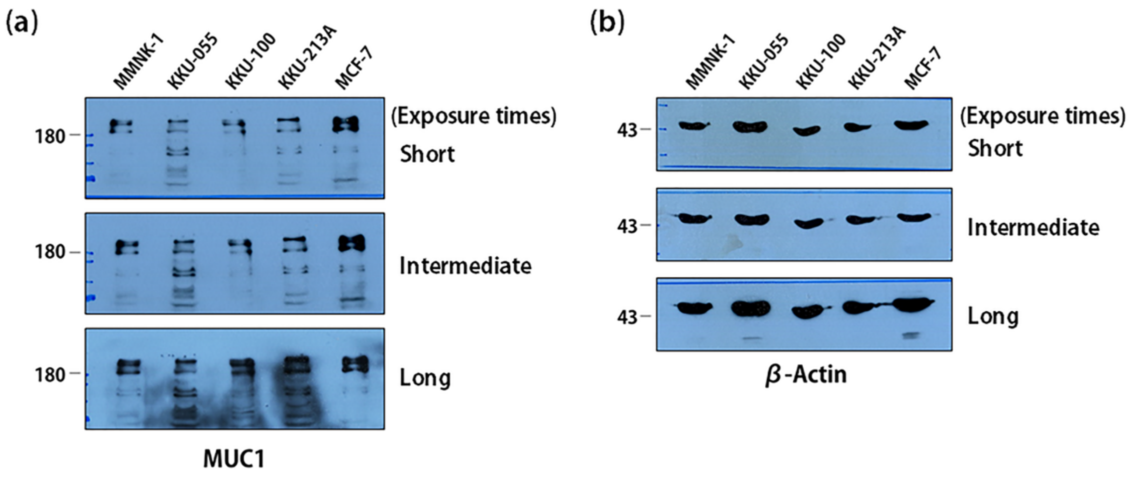

Supplementary Fig. 1. Full-length immunoblots and multiple exposure times of MUC1 and β-actin proteins in the studied cell lines. The membrane was divided into two parts for staining with (a) anti-MUC1 and (b) anti-β-actin antibodies. The membrane was exposed to X-ray films by multiple exposure times as indicated. The β-actin was used as a loading control.

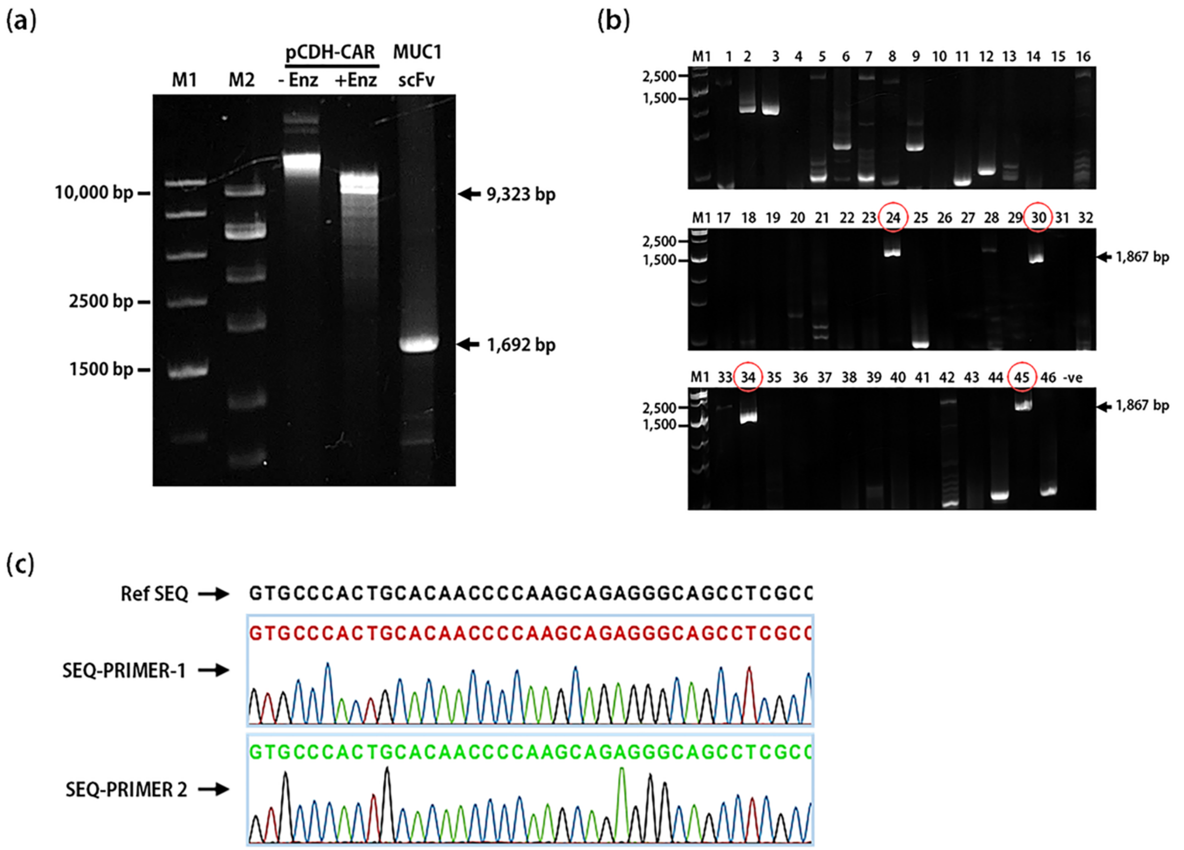

Supplementary Fig. 2. Construction and screening of anti-MUC1-CAR lentiviral plasmid. (a) Agarose gel electrophoresis of undigested (lane 3) and double-digested pCDH-CAR plasmid (lane 4), and inserted fragment (lane 5). Specific product size of digested plasmid and inserted fragment is 9,323 bps and 1,692 bps, respectively. M1 and M2 are nucleotide markers. (b) Screening of the anti-MUC1-CAR plasmid by colony polymerase chain reaction (PCR) showed the size of the specific amplified fragment to be 1,867 bps (black arrow). (c) Genotypic analysis of selected anti-MUC1-CAR plasmid by Sanger DNA sequencing compared to reference sequence (Ref SEQ) using two specific primers.

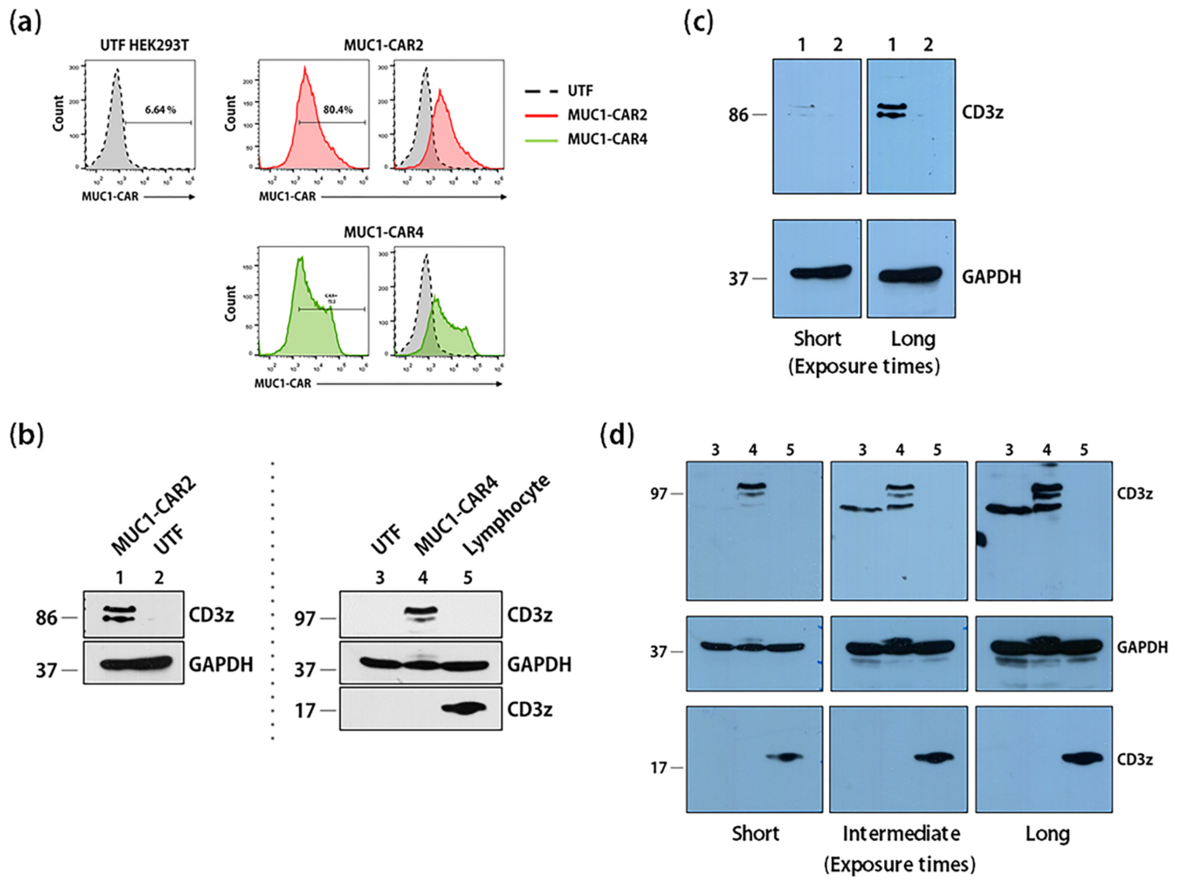

Supplementary Fig. 3. Detection of anti-MUC1-CAR2 and anti-MUC1-CAR4 expression in HEK293T cells. (a) Representative histogram of the anti-MUC1-CAR2 and anti-MUC1-CAR4 molecules expressed on HEK293T cell membrane: untransfected (gray), anti-MUC1-CAR2 (red), and anti-MUC1-CAR4 (green). (b) Detection of CD3ζ protein by immunoblots revealed the CD3ζ expression; Left panel, CAR2-transfected cells (approximately 86 kDa, lane 1), untransfected cells (lane 2); Right panel, untransfected cells (lane 3), CAR4-transfected cells (approximately 97 kDa, lane 4), and a positive control of lymphocyte lysate (approximately 17 kDa, lane 5). GAPDH, a house-keeping protein (37 kDa), was also used as an internal control. (c-d) The X-ray films with multiple exposure times as indicated: (c) anti-MUC1-CAR2-transfected; (d) anti-MUC1-CAR4.

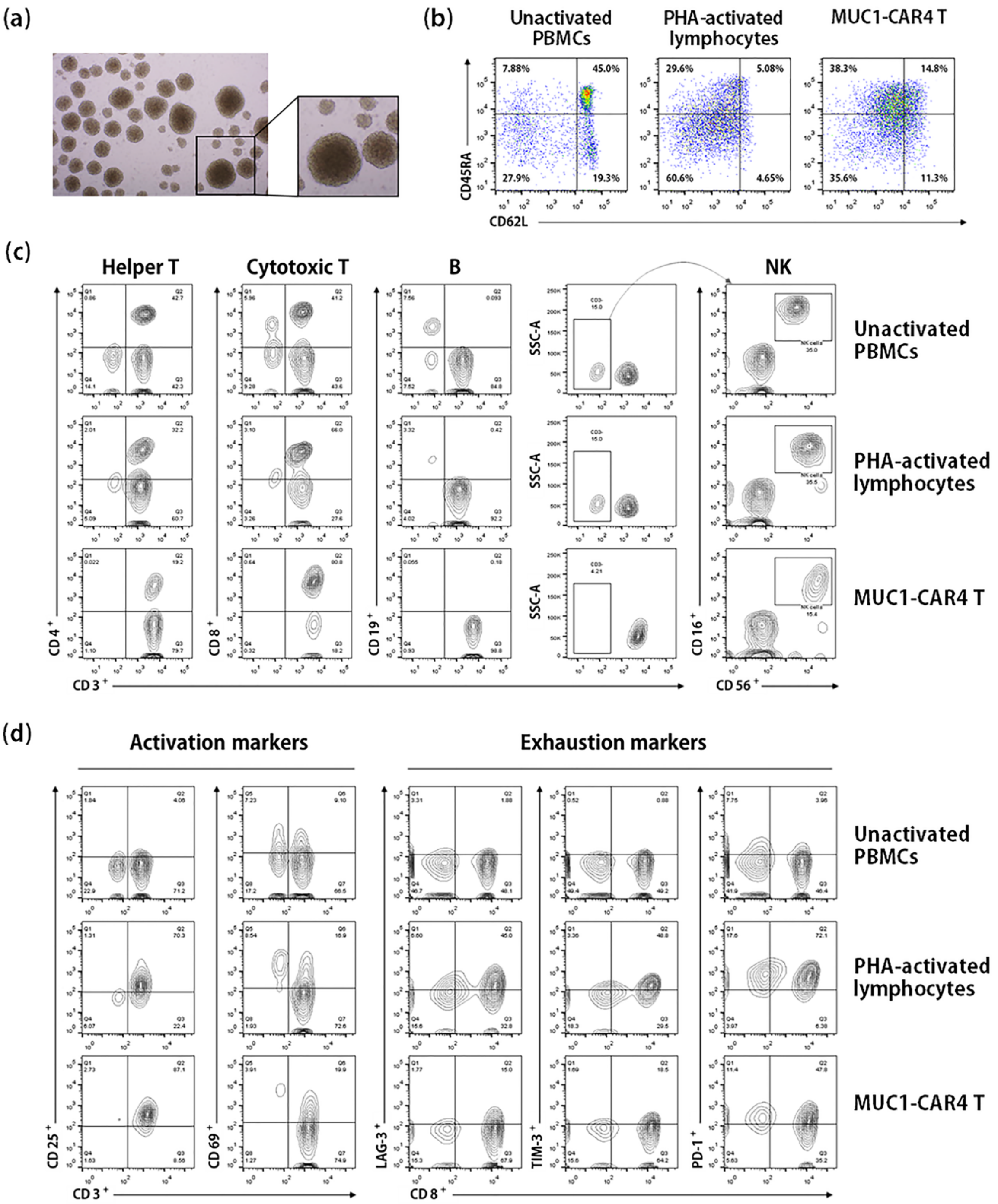

Supplementary Fig. 4. Characteristics and phenotypes of effector cells. (a) Morphology of T cells after PHA-activation. (b) Representative data and gating strategy of memory cell phenotypes: upper right, naïve T cells ($CD3^+$ $CD45RA^+$ $CD62L^+$); upper left, terminal effector T cells, T_{TE} ($CD3^+$ $CD45RA^+$ $CD62L^-$); lower left, effector memory T cells, T_{EM} ($CD3^+$ $CD45RA^-$ $CD62L^-$); and lower right, central memory T cells, T_{CM} ($CD3^+$ $CD45RA^-$ $CD62L^+$).

(c) Representative data and gating strategy of cellular phenotypes: helper T ($CD3^{+}$ $CD4^{+}$), cytotoxic T ($CD3^{+}$ $CD8^{+}$), B ($CD3^{-}$ $CD19^{+}$), and NK ($CD3^{-}$ $CD16^{+}$ $CD56^{+}$) cells. (d) Representative data and gating strategy for detection of activation ($CD3^{+}$ $CD25^{+}$ and $CD3^{+}$ $CD69^{+}$) and exhaustion markers ($CD3^{+}$ $CD8^{+}$ LAG-3^{+}, $CD3^{+}$ $CD8^{+}$ TIM-3^{+}, and $CD3^{+}$ $CD8^{+}$ PD-1^{+}).

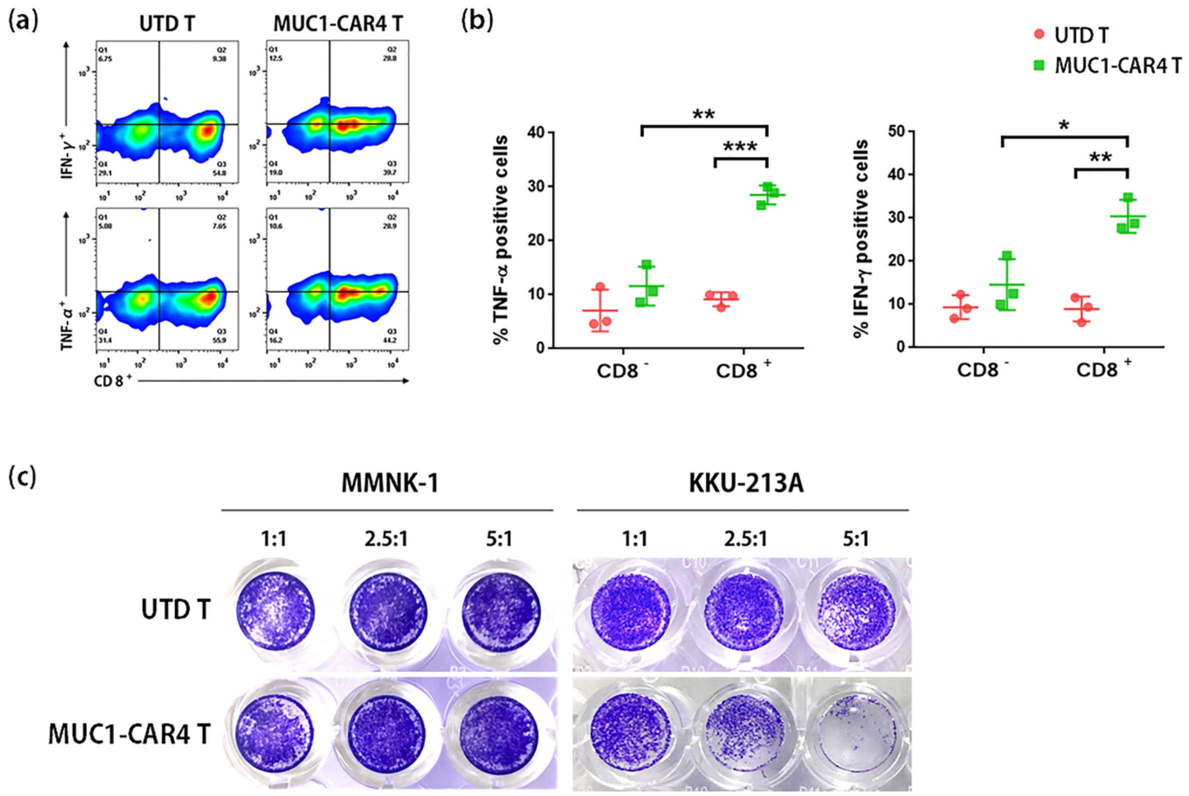

Supplementary Fig. 5. Cytokine production and killing activity of anti-MUC1-CAR4 T cells. (a) Representative data of TNF-α and IFN-γ intracellular staining in $CD8^-$ T cell and $CD8^+$ T cell populations (gated from $CD3^+$ cells) after co-culture with KKU-213A cells. (b) Summarized data presented percentages of TNF-α and IFN-γ positive cells. All data was obtained from 3 independent experiments (mean±SD), and analyzed by Student *t*-test (asterisks indicate *p*-values: $^{*}p<0.05$, $^{**}p<0.01$, $^{***}p<0.001$). (c) Crystal violet staining of viable MMNK-1 and KKU-213A cells after co-culturing with UTD T cells or anti-MUC1-CAR4 T cells at effector to target ratios of 1:1, 2.5:1, and 5:1.
